# Beyond seizures: understanding adaptive functioning in drug-resistant epilepsy in patients undergoing palliative surgery

**DOI:** 10.1007/s00381-026-07398-3

**Published:** 2026-07-17

**Authors:** Ana Valeria Duarte Oliveira, Hélio Rubens Machado, Úrsula Thomé, Geisa de Angelis, João Pereira Leite, Ana Paula Hamad, Américo Ceiki Sakamoto, Tonicarlo Rodrigues Velasco

**Affiliations:** 1 Center for Pediatric Epilepsy Surgery, University Hospital, Ribeirão Preto Medical School, University of São Paulo, Avenida Bandeirantes, 3900, Bairro Monte Alegre, Ribeirão Preto, São Paulo 14048-900 Brazil; 2 Division of Pediatric Neurosurgery, Ribeirão Preto Medical School, University of São Paulo, Avenida Bandeirantes, 3900, Bairro Monte Alegre, Ribeirão Preto, São Paulo 14048-900 Brazil

**Keywords:** Vineland scale, Pediatrics neurosurgery, Treatment outcome, Follow-up studies

## Abstract

**Purpose:**

To evaluate clinical predictors of adaptive functioning in pediatric patients with drug-resistant epilepsy (DRE) and the impact of corpus callosotomy and vagus nerve stimulation (VNS) using the Vineland Adaptive Behavior Scales (VABS) during long-term follow-up.

**Methods:**

This retrospective observational cohort study included 37 pediatric patients with DRE who underwent callosotomy (*n* = 24) or VNS (*n* = 13) at the Ribeirão Preto Epilepsy Surgery Program, University of São Paulo, between 2007 and 2018. Adaptive functioning was assessed using the VABS at three time points: preoperative, postoperative I (mean 25 months), and postoperative II (mean 51 months). Seizure outcomes were classified according to the Engel scale. Statistical analyses included analysis of variance, Pearson correlation, linear regression, and a general linear model for repeated measures.

**Results:**

All patients presented adaptive delay preoperatively, with discrepancies between chronological age and age-equivalent scores. Postoperatively, seizure reduction occurred in 70.2% of patients, with most achieving Engel class II–III outcomes. A reduction in antiseizure medication use was observed in 43.2% of patients. Adaptive functioning declined at the first postoperative assessment but gradually improved over time, particularly among patients with better seizure control. Statistical analyses demonstrated significant effects of postoperative time and seizure outcomes on adaptive functioning, with greater improvements in patients with favorable seizure control (*F* = 4.183; *p* = 0.024).

**Significance:**

Palliative surgical procedures for pediatric DRE are safe and associated with seizure reduction, decreased medication burden, and gradual improvement in adaptive functioning. Early surgical intervention may help minimize developmental decline and improve long-term adaptive outcomes.

## Introduction

Childhood epilepsy is a common neurological condition, with prevalence rates that vary widely between developed countries, ranging from 3.2 to 5.5 cases per 1000 children, and developing countries, ranging from 3.6 to 4.4 cases per 1000 children, with particularly higher rates observed in Latin America [1]. Despite treatment with antiseizure medications (AEDs), approximately one third of children fail to achieve adequate seizure control [2]. In this context, vagus nerve stimulation (VNS) and callosotomy stand out as palliative therapeutic options aimed at reducing seizure frequency and decreasing the need for medication.

Surgical intervention has become established as an effective therapeutic alternative for patients with epilepsy refractory to pharmacological treatment [3–5]. Callosotomy is indicated for patients with multifocal epilepsy, seizures with rapid generalization, or when the epileptogenic zone cannot be localized, contributing to clinical seizure control when other treatment approaches are ineffective [6, 7]. The VNS is performed as a therapeutic strategy in patients without a resectable epileptogenic focus [8]. Recent evidence, including meta-analyses, indicates that both callosotomy and VNS are effective in reducing seizure frequency [9].

Although reduction in seizure frequency is the primary goal of epilepsy surgery [10], the intervention may also provide additional benefits, including improvements in cognitive and behavioral functioning, as well as a reduction in the number of antiseizure medications used [11, 12]. Several studies suggest that surgical intervention should be considered whenever feasible [4], as recurrent seizures during childhood are associated with permanent alterations in brain function and cognitive impairment [13, 14].

The present retrospective cohort study analyzed clinical characteristics, antiseizure medication use, the presence of comorbidities, and adaptive outcomes in pediatric patients under 18 years of age who underwent callosotomy or VNS for the treatment of refractory epilepsy over a 51-month follow-up period.

## Materials and methods

### Patient selection

This study was designed as a retrospective observational cohort, conducted through a review of electronic medical records. The study population comprised 37 pediatric patients diagnosed with drug-resistant epilepsy (DRE), defined as the failure of adequate trials of two appropriately selected, tolerated, and administered AEDs regimens either as monotherapy or in combination to achieve sustained seizure freedom [2]. Between 2007 and 2018, patients underwent either callosotomy (*n* = 24) or VNS (*n* = 13). All procedures were performed at a single referral center (Ribeirão Preto Epilepsy Surgery Center, University of São Paulo) by a specialized multidisciplinary team that remained consistent throughout the study period. All participants underwent a standardized preoperative evaluation aimed at localizing the epileptic seizures.

Patients were eligible for inclusion in the study if they were older than 12 months and exhibited developmental delay, as defined by the assessment tool employed, the Vineland Adaptive Behavior Scales (VABS). Exclusion criteria included presence of psychiatric disorders in parents or legal guardians, absence of adaptive behavior assessments in any of the three study phases, prior history of epilepsy surgery, and age under 18 years at the time of the preoperative evaluation. Written informed consent was obtained from the families prior to participant enrollment. All procedures were approved by the institutional research ethics committees of the participating centers.

### Preoperative evaluation

The study was carried out by a specialized multidisciplinary team using a standardized protocol for expert assessments and complementary investigations, including video-electroencephalogram (video-EEG) and structural neuroimaging, as previously reported [15, 16].

During neurology consultations, parents or caregivers were interviewed to collect detailed clinical information, including seizure frequency, age at onset, antiepileptic drug history, family background, perinatal and developmental history, and known genetic conditions. All 37 patients underwent a comprehensive medical and neurological assessment, magnetic resonance imaging (1.5 or 3.0 Tesla), and video-EEG monitoring. Additionally, single-photon emission computed tomography (SPECT) was performed during both ictal and interictal phases to localize epileptogenic zones, using technetium-ECD as the radiotracer.

### Vineland Adaptive Behavior Scale (VABS)

#### VABS preoperative evaluation

Participants were assessed at three time points: preoperative, postoperative I, and postoperative II. Due to cognitive deficits or young age, some patients were unable to complete the full neuropsychological test battery. In these cases, the VABS were employed to evaluate adaptive functioning. The VABS is a semi-structured interview conducted with parents or caregivers, comprising 261 items across four domains: communication, socialization, daily living skills, and motor abilities [17]. Results were expressed as age-equivalent (AE) scores, reflecting the developmental performance of each patient relative to peers of the same chronological age (CA) at all study time points. Each item was scored based on task performance: 2 for “usually,” 1 for “sometimes or partially,” and 0 for “never.” Domain scores were then compared to the VABS manual reference tables to determine the corresponding AE at the time of assessment.

The researchers responsible for the statistical analysis did not participate in the administration of the VABS at any stage of the study; all assessments were conducted exclusively by the hospital’s neuropsychologists. Throughout the study period (2007–2018), the VABS was administered by more than one professional; however, the same trained neuropsychologist performed both the preoperative and postoperative assessments. Additionally, the neuropsychologist conducting the VABS remained blinded to other clinical information, including magnetic resonance imaging (MRI) results, type of surgical procedure, and seizure outcomes.

Following the preoperative evaluation and confirmation of surgical eligibility, each case was reviewed in a multidisciplinary meeting involving specialists from various fields. The purpose of this discussion was to collectively determine whether surgery should be indicated, contraindicated, or if additional invasive investigations were required to accurately map the epileptogenic zone. For cases in which surgery was approved, the procedure details, along with potential risks and benefits, were thoroughly planned and communicated. Subsequently, an orientation session was held with the patients and their caregivers, during which the team explained the decision, clearly outlined the associated risks and benefits, and addressed all questions. Only after this process did the families who agreed to proceed provide written informed consent (ICF), formally acknowledging their understanding and approval of the surgical procedure.

#### Postoperative data (phases 1 and 2)

Postoperatively, the VABS were administered at two time points: the first assessment at a mean of 25 months following surgery and the second at approximately 51 months postoperatively. All patients were followed through study completion, with no loss to follow-up. Data regarding AEDs use were collected at the time of the second assessment.

### Seizure outcomes

Seizure outcomes were evaluated using the Engel classification system [18], based on caregiver reports and review of medical records. Follow-up assessments were conducted at 2, 3, and 6 months and at 1 year postoperatively to monitor seizure control and AEDs use, allowing for ongoing evaluation and adjustment of treatment as needed. Engel classification was recorded longitudinally; for statistical analyses, only the most recent classification available in the medical records was included in the SPSS database. Engel class I comprised patients who remained seizure-free after surgery; class II included those with rare disabling seizures or near seizure freedom; class III encompassed patients with modest but clinically meaningful improvement; and class IV included those with no improvement or worsening seizure outcomes.

### Statistical analysis

Categorical variables were analyzed using absolute frequencies and percentages, whereas continuous variables were expressed as means and standard deviations, as well as minimum and maximum values, medians, and interquartile ranges. The paired *t*-test was used to evaluate the number of AEDs before and after surgery. Parametric statistics, including analysis of variance (ANOVA) using the general linear model (GLM) and Pearson’s correlation test, were employed to assess relationships between variables. To model the impact of epilepsy surgery on clinical variables, a repeated-measures GLM was applied. To evaluate the impact of changes and to determine whether any independent variable influenced postoperative outcomes, linear regression analysis was performed. Statistical significance was defined as *p* < 0.05. All data analyses were conducted using SPSS software, version 23.0 (Statistical Package for the Social Sciences, SPSS Inc., 1989–2004, Chicago, IL, USA).

## Results

### Presurgical evaluation

#### Population

Thirty-seven pediatric patients were included in the study, of whom 24 (64.9%) were male and 13 (35.1%) were female. Twenty-four patients underwent corpus callosotomy, and 13 received a VNS.

#### Clinical information

Clinical and patient characteristics related to epilepsy surgery were obtained from medical records and are reported below. The median age at epilepsy onset was 7 months (minimum: 1; maximum: 96; interquartile range [IQR]: 3, 7, 36). The median duration of epilepsy was 72 months (minimum: 0; maximum: 172; IQR: 33, 72, 109), and the median seizure frequency was 120 seizures per month (minimum: 4; maximum: 2400; IQR: 30, 120, 540). The mean CA at the preoperative evaluation was 95 ± 48 months. The mean number of AEDs was 3.2 ± 1.13 preoperatively and 2.0 ± 0.8 postoperatively.

Etiology was determined by MRI, with congenital causes being the most frequent (24 cases; 64.8%), followed by acquired causes (5 cases; 13.5%). Etiological diagnoses included malformations of cortical development in 12 cases (32.4%), unknown etiology in 8 cases (21.6%), tuberous sclerosis in 7 cases (18.9%), focal cortical dysplasia in 5 cases (13.5%), mesial temporal sclerosis in 3 cases (8.1%), and tumors in 2 cases (5.4%).

A high rate of comorbidities was also observed. According to neuropsychological reports, all 37 patients presented adaptive delay; 23 patients had a diagnosis of intellectual disability, 15 had behavioral disorders, 2 were diagnosed with autism spectrum disorder, and none of the patients reported depressive symptoms.

#### Vineland Adaptative Behavior Scale (VABS) before surgery

Our study included children with significant adaptive impairment resulting from severe DRE. In this preoperative phase, the mean VABS score was 31.0, which was lower than the patients’ CA at the same assessment, which was 95.0 months. Specifically, the mean monthly AE scores were 35.0 for communication, 42.0 for socialization, 32.0 for daily living skills, and 46.0 for motor skills.

When analyzing the degree of delay among the patients, a substantial proportion of the population exhibited a high level of adaptive impairment. Of the 37 patients, 3 (8.1%) presented with mild deficit, 8 (21.6%) with moderate deficit, 24 (64.9%) with severe deficit, and 2 (5.4%) were classified as having profound deficit. These findings confirm a substantial incidence of adaptive delay among the patients included in this study, with approximately 26 (70.3%) demonstrating developmental impairments.

A univariate analysis was conducted to investigate potential differences among etiological groups and did not reveal statistically significant differences (one-way ANOVA, *F* = 0.469; *p* = 0.498).

Regarding comorbidities, the univariate analysis did not reveal statistically significant effects of the degree of delay on comorbid conditions, including autism spectrum disorder (one-way ANOVA, *F* = 2.215; *p* = 0.125), intellectual disability (one-way ANOVA, *F* = 0.806; *p* = 0.455), behavioral disorders (one-way ANOVA, *F* = 0.372; *p* = 0.692), and depression (one-way ANOVA, *F* = 0.375; *p* = 0.544). Likewise, in our sample, the presence of AEDs did not have a significant impact on adaptive performance (one-way ANOVA, *F* = 2.091; *p* = 0.093).

A positive correlation was observed between the mean VABS score and etiology, indicating a weak association (*R* = 0.339; *p* < 0.040).

A linear regression analysis was performed using the mean VABS as the dependent variable and the following independent variables: age at seizure onset, epilepsy duration, monthly seizure frequency, etiology, and number of AEDs. Among the predictors, epilepsy duration (linear regression: *R*^2^ = 0.491; *B* = 0.521; *p* = 0.001), age at epilepsy onset (linear regression: *R*^2^ = 0.491; *B* = 0.297; *p* = 0.042), and number of AEDs (linear regression: *R*^2^ = 0.491; *B* = −0.432; *p* = 0.003) showed statistically significant associations, highlighting their importance as clinical markers in the context of severe DRE. The data are presented in Table 1.

**Table 1:** Regression analysis of VABS in relation to epilepsy clinical variables^a^

Model	Non-standard coefficients		Standardized coefficients	
	*B*	Standard model	Beta	Sig
(Constant)	23.201	6.439		0.001
**Age at onset seizures (months)**	**0.128**	**0.060**	**0.297**	**0.042**
**Epilepsy duration (months)**	**0.111**	**0.031**	**0.521**	**0.001**
Seizure frequency (months)	−0.003	0.003	−0.169	0.243
Etiology	−0.696	0.634	−0.153	0.281
**AEDs**	**−3.933**	**1.240**	**−0.432**	**0.003**

^a^Standardized coefficients

Bold values indicate statistical significance (*p*<0.05)

### Postoperative evaluation

A paired-samples *t*-test was conducted to evaluate differences in the number of AEDs between the preoperative and postoperative phases of the study. On average, the number of AEDs in the postoperative phase (mean = 2.35; SE = 0.14) was lower than in the preoperative phase (mean = 2.91; SE = 0.18), *t* = 4.742, *p* < 0.05.

#### Vineland Adaptative Behavior Scale (VABS) before surgery

All pediatric patients participating in this study underwent two VABS assessments after surgery. The mean CA at the first postoperative assessment was 113 ± 46.8 months, and the mean VABS was 21 ± 10.0 months. The mean time elapsed between the preoperative evaluation and the first postoperative assessment was 25 ± 21.0 months. The mean VABS domain scores were 25.0 months for communication, 29.0 months for socialization, 23.0 months for daily living skills, and 30.0 months for motor skills.

At the second postoperative VABS assessment, the mean time elapsed between the preoperative evaluation and the second postoperative assessment was 51 months. The mean CA of the evaluated patients was 143.0 months. During this second postoperative assessment, the mean VABS was higher, totaling 24.5 months. The results indicated the following mean scores across adaptive behavior domains: communication 28.0, socialization 34.0, daily living skills 26.0, and motor skills 33.0.

#### Postsurgical seizure outcome

The palliative surgical procedures performed included corpus callosotomy in 24 cases (64.9%) and VNS in 13 cases (35.1%). Following surgery, 14 of the 37 patients (37.8%) were seizure-free or had rare disabling seizures (Engel class I and II, respectively), whereas 23 patients (62.2%) experienced seizure reduction or no reduction in seizure frequency (Engel class III and IV, respectively).

#### Effect of seizure outcome on adaptive scores

A mixed analysis of variance was conducted to evaluate the impact of epileptic seizure outcomes on VABS across three assessment periods: preoperative, postoperative I, and postoperative II. The independent variables included time after surgery and seizure outcome, categorized according to the Engel classification (good seizure control versus poor seizure control).

**Fig. 1:**
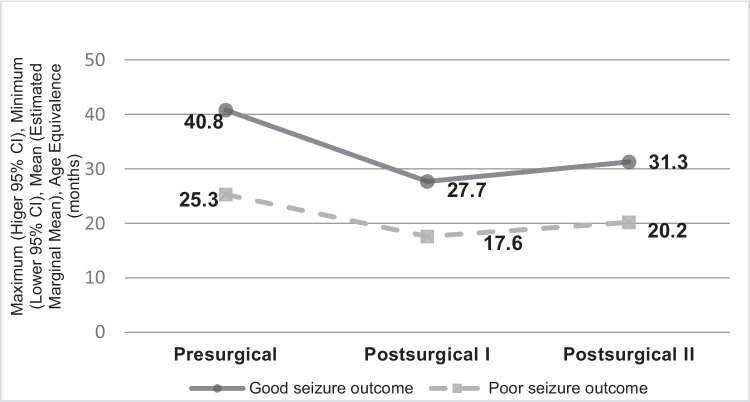
Analysis of variance assessing the impact of seizure outcomes on VABS across three follow-ups

In the between-subjects analysis, the group with good seizure control demonstrated a significantly greater improvement in VABS scores compared with the group with poor seizure control (*F* = 4.183; *p* = 0.024). As illustrated in Fig. 1, the mean AE reached 31.3 months in the group with a favorable seizure outcome (gray line), compared with 20.2 months in the group with an unfavorable seizure outcome (gray dashed line).

The within-subjects analysis revealed a significant effect of time after surgery (*F* = 29.010; *p* < 0.005), indicating a progressive increase in VABS across the three assessment periods (*F* = 17.381; *p* < 0.005). In addition, a significant effect of seizure outcome (Engel classification) on VABS was observed (*F* = 11.224; *p* < 0.005).

The potential effect of AEDs withdrawal on postoperative VABS was also examined. The model demonstrated a significant effect of surgery on VABS over time [*F* (1,277) = 17.381; *p* < 0.005], with a large effect size, accounting for 43.4% of the observed variance. Furthermore, a significant interaction between surgery and AEDs withdrawal was identified [*F* (2) = 3.046; *p* = 0.05], suggesting that the positive effects of surgical intervention on VABS were moderated by medication discontinuation.

### Callosotomy and VNS: patient characteristics and surgery-related data

Tables 2, 3, 4, and 5 summarize the main clinical and surgical characteristics of the patients included in the study. Table 2 compiles information from the preoperative phase of patients who underwent callosotomy or VNS, including demographic and clinical data. Table 3 presents the data regarding adaptive scores and comorbidities during the preoperative phase. In turn, Table 4 presents adaptive data and Table 5 data of the surgical data and information obtained at the final postoperative assessment, allowing characterization of outcomes and clinical evolution following the intervention.

**Table 2:** Clinical characteristics of all pediatric patients during the preoperative phase

	Callosotomy (*N* = 24)	VNS (*N* = 13)
Sex		
Male	18 (75%)	6 (46.2%)
Female	6 (25%)	7 (53.9%)
Age at onset (monthly)	Med: 7/Min: 1/Max: 96	Med: 4/Min: 1/Max: 72
Duration (monthly)	Med: 75/Min: 0/Max: 168	Med: 50/Min: 12/Max: 172
Seizure frequency (monthly)	Med: 217/Min: 4/Max: 2400	Med: 30/Min: 4/Max: 1590
Chronological Age (monthly)	Mean: 103.2 ± SD: 46.3	Mean: 79.6 ± SD: 48.7
Etiology		
Cortical malformation	9 (37.5%)	3 (23.1%)
Unknown	6 (25%)	2 (15.4%)
Tuberous sclerosis	6 (25%)	1 (7.7%)
Tumor	2 (8.3%)	–
Mesial temporal sclerosis	1 (4.2%)	2 (15.4%)
Cortical dysplasia	–	5 (38.5%)
Number of AEDs		
1	–	1 (7.7%)
2	11 (45.8%)	5 (38.5%)
3	7 (29.2%)	3 (23.1%)
4	3 (12.5%)	3 (23.1%)
5	2 (8.3%)	1 (7.7%)
6	1 (4.2%)	–

(Med) = median; (Min) = minimum; (Max) = maximum; (±) = average and standard deviation; (–) = absence of participants

**Table 3:** Adaptive and comorbidity characteristics of all pediatric patients during the preoperative phase

	Callosotomy (*N* = 24)	VNS (*N* = 13)
VABS (monthly)		
Communication	Mean: 30.2 ± SD: 13.8	Mean: 43.3 ± SD: 18.3
Socialization	Mean: 37.8 ± SD: 15.4	Mean: 49.7 ± SD: 16.4
Daily living skills	Mean: 29.2 ± SD: 16.1	Mean: 38.8 ± SD: 24.7
Motor skills	Mean: 44.0 ± SD: 11.4	Mean: 49.8 ± SD: 14.9
Overall mean	Mean: 27.4 ± SD: 14.2	Mean: 38.7 ± SD: 20.0
Adaptive delay		
Mild	–	1 (7.7%)
Moderate	3 (12.5%)	2 (15.4%)
Severe	14 (58.3%)	9 (69.2%)
Profound	7 (29.2%)	1 (7.7%)
Comorbidities		
Autism		
No	13 (54.2%)	12 (92.3%)
Yes	1 (4.2%)	1 (7.7%)
No information	10 (41.7%)	–
Intellectual disability		
No	–	4 (30.8%)
Yes	14 (58.3%)	9 (69.2%)
No information	10 (41.7%)	–
Behavioral disorder		
No	5 (20.8%)	7 (53.8%)
Yes	9 (37.5%)	6 (46.2%)
No information	10 (41.7%)	–
Depression		
No	14 (58.3%)	13 (100%)
Yes	–	–
No information	10 (41.7%)	–

Average and standard deviation; (–) = absence of participants

**Table 4:** Adaptive characteristics of all pediatric patients during the postoperative phase

	Callosotomy (*N* = 24)	VNS (*N* = 13)
VABS (monthly)		
Communication	Mean: 26.6 ± SD: 11.1	Mean: 31.0 ± SD: 11.8
Socialization	Mean: 30.9 ± SD: 13.1	Mean: 39.4 ± SD: 14.1
Daily living skills	Mean: 24.8 ± SD: 10.4	Mean: 28.3 ± SD: 16.0
Motor skills	Mean: 30.2 ± SD: 12.7	Mean: 40.6 ± SD: 11.5
Overall mean	Mean: 22.9 ± SD: 11.5	Mean: 27.0 ± SD: 14.0

(±) = mean and standard deviation

**Table 5:** Postoperative clinical characteristics of all pediatric patients

	Callosotomy (*N* = 24)	VNS (*N* = 13)
Number of AEDs		
1	2 (8.3%)	1 (7.7%)
2	14 (58.3%)	9 (69.2%)
3	5 (20.8%)	2 (15.45%)
4	2 (8.3%)	1 (7.7%)
5	1 (4.2%)	–
6	–	–
Engel		
I	3 (12.5%)	1 (7.7%)
II	6 (25.0%)	4 (30.8%)
III	12 (50.0%)	4 (30.8%))
IV	3 (12.5%)	4 (30.8%)

(–) = absence of participants

## Discussion

In this retrospective cohort study, we present pediatric patients with DRE who underwent corpus callosotomy or VNS over a 51-month follow-up period. The results highlight the relevance of epilepsy surgery in reducing seizure frequency and improving adaptive behavior, even in a clinically vulnerable population with severe developmental delays already present before surgery.

In the preoperative period, most patients exhibited significant developmental impairment, evidenced by a marked discrepancy between chronological age and age equivalence, with a mean difference of 32 months, indicating the deleterious impact of prolonged and uncontrolled epilepsy on neurodevelopment. Multiple linear regression identified age at seizure onset, epilepsy duration, and the number of AEDs as independent predictors of adaptive delay. In agreement with previous studies, earlier seizure onset [19, 20] and longer epilepsy duration [20] were associated with poorer cognitive performance, whereas a reduction in the number of AEDs was associated with better adaptive outcomes [19].

### Postoperative period

#### Impact of callosotomy and VNS on seizure control

Among the 24 patients who underwent callosotomy, 3 patients (12.5%) became seizure-free, while 18 (75.0%) experienced a reduction in seizure frequency; the remaining patients did not achieve significant improvement. The seizure-freedom rate observed in our sample is consistent with previous studies reporting remission rates ranging from 12.38 to 35.0%, as well as significant reductions in seizure frequency following callosotomy [7, 21–23]. In contrast, De Knegta et al. [24] reported no seizure-free patients, although a substantial reduction in seizure frequency (88–99%) was observed.

The differences noted may reflect the clinical heterogeneity of the studied populations, including factors such as epilepsy etiology, preoperative seizure burden, and the structural characteristics of brain lesions. Functional reorganization after surgery tends to occur gradually and may be limited by extensive or early-onset lesions. In addition, the high epileptic burden and the use of multiple AEDs in this cohort ranging from two to six medications per patient suggest greater disease severity. In this context, our findings reinforce the role of corpus callosotomy as a relevant palliative strategy in the management of DRE. Beyond callosotomy, VNS represents an additional neuromodulatory strategy aimed at reducing seizure burden in pediatric patients.

In our cohort, we evaluated 13 pediatric patients following VNS implantation. According to the Engel classification, 1 patient (7.6%) achieved class I, 4 patients (30.8%) were classified as class II, 5 patients (38.5%) as class III, and 3 patients (23.1%) as class IV. Overall, 69.3% of patients experienced a reduction in seizure frequency, indicating a favorable clinical response to VNS therapy. These findings are consistent with previously reported pediatric series demonstrating meaningful seizure reduction following VNS implantation [25–28]. In line with our results, Bansal et al. [29], in a large cohort of 400 patients, reported progressive improvement in seizure control over time, with a 90.5% reduction in seizure frequency at the final follow-up and seizure freedom achieved in 20.5% of patients. Taken together, these findings reinforce the importance of palliative surgical procedures as complementary therapeutic strategies in the management of pediatric DRE, contributing to the reduction of seizure burden.

#### Impact of seizure control on adaptive functioning

All patients exhibited adaptive impairments already in the preoperative period, with severity ranging from mild to severe. At the first postoperative evaluation, conducted on average 25 months after the surgical procedure, a significant improvement in seizure control was observed; however, an unfavorable effect on adaptive functioning scores was identified. The study conducted by Valova et al. [30] demonstrated that certain risk factors are directly associated with cognitive deficits and behavioral problems, including early seizure onset, longer duration of epilepsy, and polytherapy with AEDs. According to these authors, the earlier the onset of epilepsy, the longer the disease duration, and the greater the number of AEDs used, the poorer the performance across different cognitive domains. These findings are consistent with the results of the present study, although we employed the VABS as a measure of adaptive functioning, in contrast to the specific cognitive assessment tests used in previous studies.

Wang et al. [31] reported that significant improvements in adaptive skills and cognitive functioning may occur within the first 12 months following epilepsy surgery, particularly among patients who achieve complete seizure control. In contrast, other studies have not demonstrated significant improvements in intelligence quotient (IQ) or adaptive behavior. Zamponi et al. [32] investigated VABS outcomes in 60 children treated with VNS and found no significant improvement in adaptive scores. Similarly, Bodin et al. [33], in a cohort of 37 patients younger than 18 years, reported no significant improvement in IQ across the study groups. Considering the dynamic nature of adaptive development in pediatric patients with DRE, particularly in our cohort characterized by severe disease burden, it became essential to investigate whether these initial changes would persist or evolve over a longer follow-up period.

At the second postoperative assessment, conducted on average 51 months after the surgical procedure, the results of the present study demonstrate the positive impact of epilepsy surgery in patients who achieved significant seizure reduction or complete seizure freedom. During this period, a significant effect of the time elapsed between assessments was observed, indicating a progressive increase in VABS scores. Similarly, the study by Ueda et al. [34] reported that seizure-free patients exhibited greater increases in equivalent age in specific adaptive skills, particularly in the communication domain. These findings suggest that effective seizure control may facilitate improvements in adaptive functioning over time. In contrast, Viggedal et al. [35] have reported that developmental scores tend to remain relatively stable during the postoperative period, reflecting a potential plateau in cognitive trajectories following surgery.

Conversely, patients who continued to experience a high seizure frequency demonstrated minimal gains in VABS scores, even after approximately 4 years of follow-up, with a mean increase of only 2.6 months in EA. Most of these individuals already exhibited severe adaptive impairment in the preoperative period, with approximately 27% of the cohort presenting profoundly reduced VABS scores. Previous studies have reported heterogeneous findings regarding the relationship between seizure control and cognitive or adaptive outcomes. For example, Tsai et al. [36] reported that although seizure control was achieved, no significant improvements in cognitive performance were observed. These findings suggest that, in the absence of seizure freedom, the potential for adaptive recovery may remain limited. In addition, factors such as early seizure onset, high seizure burden, extensive brain lesions, and the persistence of epileptic encephalopathy may interfere with neuroplasticity mechanisms and hinder functional reorganization. Moreover, the potential effects of prolonged exposure to multiple AEDs should also be considered, as long-term pharmacotherapy may further impact neurodevelopmental and adaptive outcomes.

Over the course of follow-up, a reduction in the use of AEDs was observed in the final phase in approximately 43.2% of patients, reflecting improved seizure control and a decrease in epilepsy severity [20, 37]. In contrast, previous studies have reported that most patients maintain stable treatment regimens, with no significant differences between the preoperative and postoperative periods [38, 39]. These findings are particularly relevant given that this cohort presented a high seizure burden from the preoperative phase and further support the role of palliative surgery as an effective strategy to facilitate the gradual reduction of AEDs use.

### Limitations

The strengths of this study include its single-center design, which ensured consistency in surgical decision-making, follow-up protocols, and outcome assessment, as well as the detailed longitudinal evaluation of a cohort of patients undergoing palliative epilepsy surgery. Notably, this study incorporates adaptive functioning outcomes alongside seizure-related outcomes, with an extended follow-up period. While previous studies have primarily focused on seizure control following corpus callosotomy and VNS, data addressing adaptive functioning in this population remain limited. Importantly, participants in our cohort exhibited varying degrees of adaptive impairment prior to surgery, reflecting the severity and chronicity of their underlying conditions. This baseline impairment may have influenced caregiver- or patient-reported outcomes, potentially affecting the consistency of adaptive assessments over time. Nevertheless, the inclusion of these real-world functional outcomes provides clinically meaningful insights into the broader impact of palliative epilepsy surgery beyond seizure reduction alone.

Several limitations should also be acknowledged. The relatively small sample size, retrospective design, and heterogeneity of underlying etiologies may introduce potential confounding bias. Additionally, the prolonged use of ASMs and their potential effects on cognitive and adaptive functioning cannot be excluded. Although multivariate analyses were employed to control for selected variables, subgroup-specific factors may still have influenced the observed outcomes. Despite these limitations, the present findings contribute valuable preliminary evidence in an area with limited existing data. Importantly, this study may serve as a foundation for future prospective, multicenter investigations with larger and more homogeneous cohorts. Such studies are essential to confirm these findings and to better delineate the role of palliative surgical interventions in improving adaptive functioning and long-term quality of life in patients with DRS.

### Clinical and practical implications

The findings of this study highlight the potential benefits of surgical intervention in children with DRE. Given the observed associations among early seizure onset, longer epilepsy duration, and poorer outcomes in adaptive functioning as measured by the VABS, early identification and timely referral for surgical evaluation are critical to prevent further adaptive decline. These results underscore the importance of minimizing the duration of uncontrolled seizures during key periods of neurodevelopment, when ongoing epileptic activity may adversely affect cognitive and adaptive trajectories. Furthermore, a comprehensive preoperative evaluation, including multidisciplinary assessment and precise localization of the epileptogenic zone, is essential to maximize the likelihood of seizure control and optimize adaptive recovery. Early and effective surgical intervention may not only reduce seizure burden but also facilitate improved developmental outcomes and long-term functional independence. Collectively, these findings reinforce the importance of considering surgical treatment as part of the therapeutic strategy in pediatric patients with DRE, particularly when medical therapy fails to achieve adequate seizure control.

## Conclusions

DRE in the pediatric population is a debilitating condition associated with an increased risk of adaptive decline. In our cohort, both corpus callosotomy and VNS were shown to be safe procedures. Seizure reduction was observed in 70.2% of patients, with the majority achieving Engel class II–III outcomes. Additionally, a reduction in the need for ASMs was observed in approximately 43.2% of patients, accompanied by adaptive levels indicating a transient decline in performance during the immediate postoperative period. Over the course of follow-up, greater adaptive recovery was observed, particularly among patients with better postoperative seizure control. However, the increasing discrepancy between CA and AE highlights the importance of early surgical intervention to optimize developmental outcomes. Prospective studies with larger samples, socioeconomically diverse populations, and longer follow-up are needed to confirm these findings and to improve long-term adaptive outcomes in this population.

## Data Availability

The data that support the findings of this study are not openly available due to reasons of sensitivity and are available from the corresponding author upon reasonable request.
